# Moral underdetermination and a new skeptical challenge

**DOI:** 10.1007/s11229-022-03529-w

**Published:** 2022-05-06

**Authors:** Marius Baumann

**Affiliations:** EthikZentrum für Ethik und Philosophie in der Praxis, Ludwig-Maximilians-Universität München, Geschwister-Scholl-Platz 1, 80539 Munich, Germany

**Keywords:** Moral underdetermination, Skeptical arguments, Derek Parfit, Consequentializing

## Abstract

In this paper, I introduce a new challenge to moral realism: the *skeptical argument from moral underdetermination*. The challenge arises as a consequence of two recent projects in normative ethics. Both Parfit (On what matters, vol 1. Oxford University Press, Oxford, 2011a) and a group called *consequentializers* have independently claimed that the main traditions of normative theories can agree on the set of correct particular deontic verdicts. Nonetheless, as Dietrich and List (Philos Rev 126(4):421–479, 2017) and myself (Baumann in J Ethics Soc Philos 13(3):191–221, 2018; Australas J Philos 97(3):511–527, 2019; Ethical Theory Moral Pract 24(4):999–1018, 2021a) have argued, the traditions still disagree about why these are the correct verdicts. This means that we can understand the situation in terms of an idea from the philosophy of science, the underdetermination of theory by the evidence. Yet underdetermination figures in one of the most important skeptical challenges to scientific realism. I show how an analogous skeptical argument can be construed for the moral realm. I propose a standard form for that argument. I then defend it against three possible objections, arguing that it is at least as plausible as, if not more plausible than, its counterpart in the philosophy of science.

## Introduction

Traditionally, differences in people’s moral views have figured prominently in challenges to moral realism. Most influential has been the so-called *argument from disagreement* that tries to cast doubt on the idea that a field so pervaded by disagreements, as is allegedly the case in ethics, could allow for genuine knowledge and truth.^1^ Realists should therefore have rejoiced when, independently of each other, two recent projects in normative ethics promised to show that the differences between our main moral traditions can be resolved. Parfit (2011a) has argued that the best versions of three of the most important traditions actually agree on what matters, that is, they agree on a set of principles and deontic verdicts. So-called *consequentializers* have gone a step further and claimed that for *any* nonconsequentialist theory they can come up with a consequentialist counterpart which is deontically equivalent.^2^ If correct, these projects seem to deal a decisive blow to the argument from disagreement and thereby, indirectly, to strengthen moral realism.

I argue that realists should curb their enthusiasm. As Dietrich and List (2017) and myself (Baumann 2018, 2019, 2021a) have proposed, Parfit’s and the consequentializers’ results are best understood in terms of a phenomenon known from the philosophy of science: the underdetermination of theory by the evidence. Just as theoretically incompatible scientific theories can sometimes account equally well for the empirical data, incompatible moral theories can sometimes account equally well for our deontic verdicts. This is a highly interesting result in its own right. However, what is most remarkable for our purposes, is that underdetermination standardly figures in antirealist arguments in the philosophy of science. Indeed, it is often taken to lead to one of the two most important challenges to scientific realism.^3^

The aim of this paper is to show how a structurally analogous skeptical argument can be construed and defended for the moral realm. I start by tracing the recent developments in normative ethics that set the stage for the new challenge. Next, I explain how a structurally analogous situation in science has given rise to a challenge: the skeptical argument from underdetermination. I then propose how an analogous argument can be construed for the moral realm. Finally, I defend the argument against three possible objections, arguing that it is at least as plausible as, if not more plausible than, its counterpart in the philosophy of science.

A couple of caveats are in order before I begin. First, I neither provide a defense of the two projects in normative ethics, nor of the thesis of scientific underdetermination. Instead, my aim is the rather restricted one of showing (i) how a new skeptical challenge arises *provided* Parfit and the consequentializers are correct in their claims about normative ethics and (ii) that this challenge is at least as strong in ethics as it is in science.

Second, since the argument is of the skeptical variety, it is (primarily) directed against versions of moral realism that include an epistemic component, that is, a claim to the effect that we can know (at least some) moral truths. Some moral realists may want to avoid this commitment and restrict themselves to the purely metaphysical claim that there are moral truths. These realists will therefore not feel threatened by the new skeptical argument, just as they presumably weren’t bothered by the older argument from disagreement. It would go beyond the limits of this paper to argue against such an understanding of moral realism.^4^ However, I will, in Sect. 4.2, offer some considerations why such an exclusively metaphysical position seems at least less attractive in the ethical than the scientific domain. Considering the importance of the underdetermination argument in the philosophy of science, the moral version of this argument should thus be of general interest nevertheless.

Third, there is a crucial disanalogy between the definitions of scientific realism and moral realism which impacts the structure of the argument. In the philosophy of science, the issue of realism versus antirealism is generally framed likes this: realists and antirealists agree that there are facts about, and we can often have knowledge about, observables. Where they differ is whether we should also believe in what our theories tell us about unobservables. Thus, to be considered a scientific realist, one has to buy into the theoretical claims our theories make.^5^ In metaethics, the dialectical situation is different. Here, realists traditionally only claim that there are *some* moral truths (of which we can have knowledge) whereas antirealists in turn claim that there are no such truths (and we can hence not have such knowledge at all). The antirealist position in ethics is thus stronger than the one in science. Moral antirealists, at least so it would seem, have to deny not only the theoretical explanations of why some acts are right or wrong, but also that there are some acts which are right or wrong (and which we can have knowledge of). For the most part of the paper, I will be concerned with arguing for the first part of this charge, that is, why we should withhold belief in our moral theories. Only in Sect. 4.3 will I be able to tackle the question of (knowledge about) truths regarding which actions are right or wrong.

## Setting the stage: two recent developments in normative ethics

### The received view and its challengers

In normative ethics, a number of moral theoretical traditions, such as Kantianism, consequentialism, and contractualism, take center stage. As the received view has it, these traditions are mutually incompatible. They offer rival accounts as to what should be done as well as why it should be done. Put slightly more technically, the received view holds that the main moral traditions disagree regarding the sets of particular deontic verdicts they yield, that is which specific acts are right or wrong, obligatory, forbidden, or allowed, as well as regarding the explanations or reasons they put forward for why a specific act has its deontic status.^6^

Although there have been the occasional heretics,^7^ it is safe to say that this view has been dominant for a long time, and not without a reason. After all, consequentialists focus on evaluating the outcomes of acts, Kantians give special weight to autonomy or the good will, and contractualists highlight what can or cannot be reasonably accepted or rejected. Surely, these different starting points should lead to different normative outcomes? Furthermore, the received view accords well with everyday normative theorizing, which in large parts consists in showing how one moral tradition is superior to another insofar as it can better account for our intuitions about specific cases.^8^

Notwithstanding its initial support, however, the received view has recently come under sustained attack from two projects. The first one is due to Derek Parfit. In his 2011 *On What Matters*, Parfit aims to show that the best versions of three of the most important families of moral theories, namely Kantianism, consequentialism, and contractualism, actually agree about what is right and wrong.^9^ His reasoning for this surprising conclusion goes roughly like this. Parfit first analyses the three traditions in great detail and identifies what he sees as their best versions.^10^ Next, he comes up with an ingenious argument, the *Kantian argument for Rule Consequentialism*.^11^ What this argument aims to show, is that those principles that everyone can rationally will are also the ones which, if universally accepted, would make things go best. The former principles are the ones we should chose on Parfit’s preferred version of Kantianism. The latter follow according to his preferred version of consequentialism. Therefore, if the argument is correct, Kantianism implies consequentialism. Similarly, Parfit thinks the same principles are also the ones that no one can reasonably reject, which makes them correct on Parfit’s preferred version of contractualism. Taken together, the argument, if successful, thus shows that the best versions of the rival traditions do not disagree when it comes to their principles. Since the set of deontic verdicts a theory yields follows from these principles, the traditions therefore agree about all their deontic verdicts.

The second project is more ambitious still. A number of philosophers have recently argued that they can *consequentialize* any nonconsequentialist theory.^12^ What they mean by this is that they can account for the exact deontic verdicts of any plausible nonconsequentialist theory by construing a consequentialist counterpart theory. Witness a typical characterization:

*Deontic Equivalence Thesis (DET)*: [...] [F]or any remotely plausible nonconsequentialist theory, there is a consequentialist counterpart theory that is deontically equivalent to it such that the two theories are extensionally equivalent with respect to their deontic verdicts. (Portmore 2011, p. 85)

The recipe to consequentialize is fairly simple: we first identify whatever moral requirements are relevant to the target nonconsequentialist theory. We then reinterpret these requirements as value judgements about the consequences of certain (classes of) acts. Finally, we only need to weigh (the value of the consequences of) these acts according to a tailor-made theory of the good, in order to yield the same deontic verdicts for a consequentialist counterpart theory. Over time, consequentializers have made considerable progress identifying which features of nonconsequentialist theories would have to be accounted for as well as making suggestions as to how this could be done.

At this stage, it remains to be seen whether the two projects will ultimately prove to be successful. However, as I already explained, I am not going to evaluate the cogency of the projects in normative ethics, thus we don’t have to delve deeper into the details. Instead, I want to turn away the attention from the implementation of the two projects now and have a closer look at their interpretation.

### The underdetermination interpretation

Interpretations of the two projects vary substantially. Parfit thinks of his own project as one of reconciliation. He explicitly argues that none of the traditions is to be privileged and that we should combine insights from all of them.^13^ Consequentializers’ outlook, in contrast, is more reductionist than reconciliatory. Some believe that their project entails that there is only one tradition after all, consequentialism.^14^ Others hold that consequentializing shows the rival traditions to be merely notationally different.^15^ Finally, one opponent of consequentializing has argued that the project, if successful, would prove consequentialism to be an empty tradition.^16^

These differences notwithstanding, however, all of the interpretations above see the two projects in stark opposition to the received view of the moral traditions. On none of them is it true that the main traditions should be thought of as competing alternative frameworks. Yet this shared heretical thrust has not been noted immediately.^17^ Only lately has a unified framework to understand the two projects gained some traction: the *underdetermination interpretation*.

That interpretation is first offered as a side note to an attempt to provide a new formalization of moral theories. Inspired by decision-theoretic work, Dietrich and List present what they term the *reason-based representation* of moral theories, which differentiates between theories along two dimensions:

Reason-based representations encode not only a theory’s *action-guiding recommendations* (i.e., how we should act, according to the theory), but also the *reasons* behind those recommendations (that is, why we should act in that way). (Dietrich and List 2017, p. 422)

The reason-based representation helps us to (formally) understand the crucial distinction between, on the one hand, the set of deontic verdicts a theory yields—the theory’s deontic content—and, on the other hand, the theory’s explanatory framework—its reasons structure. Thinking in terms of this distinction, Dietrich and List argue, highlights an under appreciated phenomenon:

[...] different moral theories may coincide in all their action-guiding recommendations, despite arriving at them in different ways. (Dietrich and List 2017, p. 422)

They call this phenomenon the *underdetermination of moral theory by deontic content*. The allusion is of course to the famous thesis of *underdetermination of scientific theory by the evidence*. Following the work of Duhem (1906) and Quine (1951), philosophers of science have debated a highly influential idea: sometimes more than one scientific theory is able to account for the evidence.^18^ At the same time, the rival theories may still offer incompatible explanations. They are, in other words, *empirically equivalent and logically incompatible*.

Dietrich and List argue that there is a similar phenomenon to be found in ethics. Just as two or more rival scientific theories can sometimes account equally well for all the evidence at hand, two or more rival moral theories might account equally well for the same set of deontic verdicts. Instead of being underdetermined by the empirical data, moral theories can be underdetermined by their deontic content. To be clear, the idea of deontically equivalent yet theoretically incompatible theories had already been proposed as an interpretation for both Parfit’s as well as the consequentializers’ projects.^19^ What distinguishes Dietrich and List’s contribution, is that they combine these insights within a common (formal) framework and note the analogy to the philosophy of science.

The underdetermination interpretation has two crucial advantages over rival interpretations of the two projects.^20^ First, it does not rely on a impoverished picture of the functions of moral theories. For why, we can ask with regards to consequentializing, should the fact that different theories lead to the same verdicts show that they are the same theories or only notationally different? This would only follow if deontic adequacy was all that moral theories aim for, which is obviously not how most ethicists understand them. Additionally, we can ask why Parfit’s arguments should suffice to reconcile the rival traditions? After all, he has at most shown that the best versions can arrive at the same verdicts. Yet the traditions still give mutually incompatible explanations for why those are the correct verdicts.^21^ Second, allowing for these differences in explanation also help us make better sense of the fact that the main moral traditions have, for the longest time and by most philosophers, been thought to stand in stark contrast. Being compatible with a fuller picture of the functions of moral theories as well as being able to make better sense of the antagonistic understanding of the moral traditions inherent in the received view are clear advantages of the underdetermination interpretation. We should thus prefer it, I think, pending further arguments to the contrary. Yet this, or so I will argue in what follows, has far-ranging metaethical repercussions.

## The new skeptical challenge

### Scientific underdetermination and skepticism

Dietrich and List remain mostly silent on possible metaethical upshots of moral underdetermination.^22^ This is also largely the case when it comes consequentializers, who are mainly interested in how their project impacts our picture of normative ethics.

Parfit, in contrast, is very aware of the metaethical impact that his project might have. He considers the fact that our best theories agree on what matters to be good news for moral realism.^23^ Since disagreements about our moral verdicts are exploited by antirealists, surely realists should welcome the recognition that, at least when it comes to our best theories, there are no such disagreements left?

I disagree. Taking inspiration from the philosophy of science, I have argued that Parfit’s argument backfires. To see why, consider the following argument:

1. If two moral theories (MT) can account for exactly the same evidence, it is equally reasonable to believe either of them.
2. If it is equally reasonable to believe either of two MT, we have no reason to attribute truth to one but not the other.
3. If two MT contain incompatible propositions, they cannot both be true.
4. If two MT cannot both be true, and we have no reason to attribute truth to one but not the other, then none of them should be considered true. UMT. There are alternatives to even our best moral theories that can account for exactly the same evidence while containing incompatible propositions.
5. Therefore, even our best moral theories should not be considered true. (Baumann 2018, p. 208)

This, as far as I know, is the first explicit adaption of the skeptical underdetermination argument from science to ethics. However, the argument is tailor-made to counter Parfit’s reasoning and this brings with it limitations when it comes to a more general application.^24^

What I want to do next, therefore, is to propose a version of the skeptical challenge that is both more general and more simple. It is more general because it is an argument against a wide variety of moral realisms, not just Parfit’s version. It is more simple because it is stripped down to the most basic premises as they can be identified in the philosophy of science.

Underdetermination has figured in skeptical arguments in the philosophy of science starting right with Duhem’s classical treatment of the topic. Nowadays, the skeptical argument from underdetermination is often seen as one of two main arguments against scientific realism.^25^ As one would expect, different reconstructions of the argument have been proposed. Yet it is possible to identify a core structure of the argument.^26^ That structure is as follows:

1. *Extensional Equivalence*: There are empirically equivalent rivals to even our best scientific theories.
2. *Evidential Equivalence*: Some of those empirically equivalent rival theories are equally believable as the original theories.
3. *Withholding of Belief*: When facing two equally believable rival theories, belief should be withheld.
4. Belief in even our best scientific theories should be withheld.

As mentioned, I will not defend the argument as it pertains to the scientific realm. However, I want to make a few comments about the premises before I transfer the argument to the moral realm. (P1) is a specific version of an underdetermination claim. Two theories are *empirically equivalent* if they entail the same set of propositions about the evidence. They are *rivals* if they cannot be true at the same time.^27^ Most commentators agree that this premise is uninterestingly true. The reason for this is that (P1) does not presuppose that the rival theories are even remotely plausible. It is easy to come up with algorithms to produce empirically equivalent yet entirely *bizarre* rivals to any number of scientific theories.^28^

(P2) states that some of the rival theories are equally believable. As we will see, this claim comes down to the prediction that no factors other than the data will decide the case for one of the theories. To establish (P2), antirealists in science can basically use two strategies. They either flat out deny that anything beyond extension is relevant to the truth of a theory or they maintain that any additional theoretical virtues, although relevant to the truth, somehow fail to tip the balance. On the first option, all empirically equivalent theories come out as equally believable. On the second option, this need not be the case, as long as there is at least one rival which comes out as equally believable. We will come back to these two options.

(P3) asserts that the appropriate reaction to equally believable, yet rival, theories is to withhold belief. This introduces the skeptical element. Since skepticism is an epistemological position, it follows that the underdetermination argument is only a threat to scientific realism if that position does itself include an epistemic component. Most philosophers of science do accept that there is at least a weak epistemological component to scientific realism.^29^ Chakravartty even takes the epistemological component to be the most important one, when he characterizes scientific realism as:

[...] [A] positive epistemic attitude toward the content of our best theories and models, recommending belief in both observable and unobservable aspects of the world described by the sciences. (Chakravartty 2017, p. 2)

The skeptical argument from underdetermination is, of course, by no means universally accepted and has prompted many sophisticated replies. I consider some of these, or rather their counterparts in ethics, below. However, before complicating matters any more, I first want to adapt the argument to the moral realm.

### Adapting the skeptical argument to the realm of ethics

To adapt the skeptical argument from the scientific to the the moral realm, we substitute *deontic* for *empirical* and *moral* for *scientific*. This yields:

1. *Extensional Equivalence*: There are deontically equivalent rivals to even our best moral theories.
2. *Evidential Equivalence*: Some of those deontically equivalent rival theories are equally believable as the original theories.
3. *Withholding of Belief*: When facing two equally believable rival theories, belief should be withheld.
4. Belief in even our best moral theories should be withheld.

Merely transferring the skeptical argument in this way obviously does not prove much for two kinds of reasons. First, some of the objections leveled at it in the philosophy of science might well be applicable in ethics as well. Second, the argument might fail for reasons that have to do with the specific nature of ethics.^30^ I will consider objections of both kinds in Sect. 4. Before I do that, however, I want to make a few remarks about the analogy and the adaption of the argument.

To start, it is important to clarify what the analogy between the scientific and the moral presupposes regarding the metaphysical as well as the epistemological status of the two domains. Dietrich and List don’t go much into the details of this, since it arguably doesn’t make much of a difference when it comes to formal representations of theories. I have suggested that what’s presupposed is only a *structural* similarity.^31^ The claim is not that there is underdetermination in ethics *because* there is underdetermination in science and ethics and science are sufficiently similar in their nature. For the analogy to work, empirical data and deontic verdicts need not be on par epistemically or metaphysically *in every respect*. Instead, all that is required is a similarity in the *structure* of the relationship between theories and their relata.

However, there is a disanalogy between the two realms precisely when it comes to the relationship between theories and data. In science, we typically assume that theories make predictions and these are then tested against the background of the available data. In the proposed analogy, it might seem that the deontic verdicts that follow from moral theories take the place of the predictions in science. But this would mean that we are missing the analogs to the empirical evidence in science, making it impossible to test the correctness of of the moral verdicts that our theories yield. We might therefore fear that the analogy does not even start to make sense. However, I think that there are at least two ways to answer this worry. The first one would be to introduce the notion of moral intuitions, or considered judgements, which could take the place of the data of morality, to which the deontic verdicts that follow from moral theories need to correspond. Several people have suggested to think of the analogy between science and ethics in this vein and I have no quarrel in principle with amending the analogy accordingly.^32^ However, it might also make the analogy more controversial, since the notion of moral intuitions is itself debated.^33^ In addition, it makes it more complicated to integrate the present proposal into the current literature on consequentializing, which makes no use of the notion of moral intuitions, instead only referring to the deontic content of theories. I therefore propose a second answer, which is to acknowledge that the analogy is not perfect, but should still do its purpose. This is the case because underdetermination, as Douven points out, at is core concerns the epistemic relationship between just two distinct classes of propositions.^34^ One class underdetermines a second class if knowledge (or justified belief) of all the members of the first class is not sufficient for knowledge (or justified belief) of the members of the second class. This is the case if, following Dietrich and List, we consider the deontic content and the reasons structure of a theory. The class of all the particular deontic verdicts that make up the deontic content of moral theories is not sufficient to decide upon one reasons structure. The reasons structure is therefore underdetermined by the deontic content of a moral view.

Apart from these remarks on the analogy, note also some of the particularities of the framing of the skeptical argument. First, why is the argument framed in terms of belief in *theories*? This might strike some as odd, since philosophers of science often tend to express the idea of underdetermination in terms of unobservable objects. What is challenged is just our belief in the unobservable objects that scientific theories postulate in order to explain what we can readily observe. The problem is that the observable versus unobservable distinction has no obvious application in ethics, so we can’t express the argument in these terms.^35^ Yet, as it is widely acknowledged that the phenomenon of underdetermination is ultimately not restricted to the scientific domain,^36^ it should be possible to express it in a way that does not presuppose this distinction. I propose that framing the problem in terms of belief in theories is one such way and I will have to say more about how this framing affects the general argument in Sect. 4.3. Second, one might wonder why the argument is expressed in terms of *belief* in theories. This presupposes that realism entails an epistemic component. As we saw, this has seemed plausible to many scientific realists and one can easily find prominent moral realists who explicitly accept the component, too.^37^ Nevertheless, the epistemic component is no necessary part of moral realism, rendering its inclusion controversial. I will come back to this issue in Sect.  4.2. Until then, I have to ask readers’ patience with what might seem to them a somewhat idiosyncratic framing of the skeptical argument.

Finally, note also that the argument only serves the negative purpose of presenting a challenge to scientific realism. It makes no mention of what the alternative could be. Underdetermination in science has been connected to a variety of views, such as *conventionalism*, *instrumentalism*, *social constructivism*, or *constructive empiricism*.^38^ If one wanted to construct a positive argument for a specific kind of moral antirealism, one would need to be more precise in this respect. However, the goal here is not to defend a specific antirealist position but only to defend a specific argument against moral realism. To this end, I now turn.

## In defense of the skeptical argument

In what follows, I am going to defend the argument against three possible objections. I do not discuss objections to (P1*). On the one hand, this would take us too far into the details of the two projects in normative ethics, which I can’t do here. On the other hand, and more importantly, I think that philosophers of science are right that (P1) is uninterestingly true and the same is the case for (P1*). It is not difficult to come up with a deontically equivalent alternative to any moral theory unless we specify minimal conditions of plausibility. I do consider two objections to (P2*) and (P3*), respectively, which are inspired by similar objections in the philosophy of science. In addition, I discuss one more objection that is specific to ethics or, more precisely, the dialectical situation that we face in ethics. I do not claim that these objections exhaust realists’ options, although I do think that they are among the most important. I also do not claim to be able to definitively counter the objections. Instead, what I want to show is that the antirealist argument is at least as plausible in the moral domain as it is in the scientific. Since the skeptical argument from underdetermination is considered one of the most important challenges to realism in the philosophy of science, showing that the situation is at least equally promising for antirealists in ethics should go a long way towards establishing the relevancy of the argument in ethics.

### Objection I: Against evidential equivalence

Let us start with the objection to (P2*). Is it true that (at least some of) the extensionally equivalent rivals to our best moral theories are equally believable? Not necessarily, some of the opponents of the skeptical argument in science have claimed. They take issue with the idea that the empirical data alone can adjudicate between rival theories. This, to them, betrays a much too simplistic picture of scientific methodology. Scientists not only look at what predictions are deductively entailed by a theory but also take into account further criteria, so-called *theoretical virtues*. Beyond their predictions, theories can differ when it comes to their simplicity, their matching with other theories, etc. The deductively deducible consequences of a theory can thus not be identified with its evidential support, since the latter involves further criteria. If these further criteria tip the balance for one theory, the skeptical argument fails and we are in such a case justified in believing that theory.^39^

Is the skeptical argument thereby repudiated? Again, the answer is, *not necessarily*. I have already alluded to two possible counters for antirealists. One is to tackle the objection head-on, by denying that theoretical virtues are relevant to the truth of a theory at all. The most famous proponent of this strategy is van Fraassen (1980). Virtues such as simplicity, van Fraassen claims, provide us with pragmatic criteria for theory choice at best, but have nothing to do with a theory being true.^40^ They thus cannot help us to decide which of two empirically equivalent theories is the correct one. The other, less direct, strategy for the defender of the skeptical argument is to cast doubt on whether the theoretical virtues will indeed be sufficient to decide between theories on a case by case basis. Thus, even if the theoretical virtues are indicative of the truth of a theory, it might still be that different virtues are exhibited by different theories. Those virtues might prove to be of equal weight, or, as Tulodziecki proposes, they might even turn out to be incommensurable.^41^

It is not difficult to see how these considerations are relevant in ethics as well. Moral realists could argue that just as empirical equivalence must not be identified with evidential equivalence, so shouldn’t deontic equivalence. Moral theories exhibit theoretical virtues as well, and those might tip the balance in favor of one of the rival traditions.^42^ Given this, how should antirealists in ethics defend (P2*)?

The two strategies are not combinable since one accepts that theoretical virtues are relevant to the truth of a theory whereas the other denies it.^43^ Antirealists thus have to make up their mind. I propose that they should opt for the second, less aggressive, strategy. To appreciate why, we should ask why antirealists in science are driven to such drastic measures as claiming, for example, that the simplicity of a theory has nothing to do with its truth, in the first place. I propose that the main reason proponents of the skeptical argument in science are tempted to flat out deny the relevance of theoretical virtues is the sheer number of theories at play. If scientific antirealists did not deny in principle that theoretical virtues might break the tie, they would have to prove their ineffectiveness for all the best scientific theories in isolation. That means showing how there are empirically equivalent theories to all the best theories only to then also having to show that, in each of these cases, the additional theoretical virtues do not decisively prefer one of the theories. Instead of taking on that Sisyphean task, it is tempting to deny the importance of theoretical virtues in principle. However, this is a highly controversial move, especially considering the importance that many scientists do in fact accord to ampliative forms of reasoning, such as *inference to the best explanation*.

Do proponents of the skeptical argument in ethics really want to make an analogous claim in ethics, to the effect that no matter how perfectly simple and elegant a theory is, that does not render it in any way more plausible than some highly complicated, ad-hoc theory? It seems clear that they are better off not having to commit themselves to such a controversial claim.^44^ Fortunately for them, there is less pressure to accept this claim in ethics. The reason for this is that there is a crucial difference in the scope of scientific and moral theories. Scientific theories are characteristically *local* theories, meaning that they account for different subsets of the evidence in different domains (biological theories in biology, chemical theories in chemistry, and so on). In contrast, moral theories are typically considered to be *global*. They cover the whole realm of deontic verdicts (or something close enough).^45^ This means that when it comes to the moral domain, all that has to be shown is that our one best moral theory has a deontically equivalent alternative that is equally well supported by additional virtues. That is certainly more easily done than showing the same thing for all (or most of) the best theories of science.

How likely is it, then, that the best moral theory has at least one deontically equivalent rival that is also equally believable? We can not say for sure at this stage, since there is no consensus on which the best moral theory is. However, we can make an informed guess. First, as Schroeder points out, weighing against each other some of the theoretical virtues that have been claimed for the competing moral traditions might prove to be extremely difficult.^46^ For example, consequentializers have claimed that their theory has the advantage of being compatible with the so-called *compelling idea*, i.e., the idea, that it cannot be wrong to do what would have the best outcomes. Nonconsequentialists have countered that even if consequentialists can account for all the right verdicts, they are nevertheless offering the *wrong kind of reasons*.^47^ To me, it is not clear how we would even start to determine the relative weight of such considerations, hinting at a possible case of incommensurability.^48^ Second, note that even one of the staunchest defenders of underdetermination in science, Kukla (2001), agrees that the empirically equivalent alternatives he comes up with are *bizarre*.^49^ In contrast, Parfit has to assume that his three favorite theories are equally plausible (otherwise he would have to prefer one of them) and defenders of consequentializing think that their theories are at least as plausible as the deontological counterparts. Thus, when it comes to the evaluation of the plausibility of the rival theories by the people who have proposed them, ethicists seem to be more optimistic.

Additionally, if it is indeed the case that moral theories cover the whole realm of moral verdicts, that also means that we are facing what is sometimes called a *permanent* form of underdetermination. This is in contrast to many cases in science. Recent discussions of scientific underdetermination have emphasized that in many of the historical examples the rival theories were only temporarily underdetermined, meaning that when new data became available, it turned out that the theories actually made different predictions and so could no longer be considered empirically equivalent. Stanford (2001) has therefore argued that we should think of underdetermination as a transient, although recurring, phenomenon. Ethics might be different in this regard. If moral theories do indeed cover the whole realm of deontic verdicts, meaning that their principles apply not only to past and present cases, but also to scenarios that might only become relevant in the future (or to thought experiments that have not already been conceived), it would point to a permanent form of underdetermination. This would considerably strengthen the relative plausibility of the skeptical argument in ethics, since many scientific realists reject the skeptical argument precisely because underdetermination often proves to be of a transient nature. Moral realists might not have this option.^50^

We can thus quite confidently say that when it comes to the first kind of objection, antirealists in ethics are in an equally good, if not a better, position than antirealists in the philosophy of science.

### Objection II: Against withholding of belief

Still, why be so skeptical? Is the reasonable reaction to facing two incompatible but equally well confirmed theories really that we should withhold belief in any of them, as (P3*) suggests? Some philosophers of science, when facing the analogous question, have argued that it is not. To them, this seems an overly hasty reaction when there is a perfectly acceptable alternative. Instead, we could simply accept that we do not have, and possibly never will have, the evidence to adjudicate between the competing theories, but nonetheless insist that there is a fact of the matter about which is true, independently of our ability to ever come to know of it. We are thus free to believe that only one theory is true, even if we can not say which.^51^

Is this a viable option in ethics, as well? At least in principle, is seems so. If realists in science can claim that their justification in believing in the truth of their theories transcends the evidence, then why should realists in ethics not be entitled to do so?

We have at this stage arrived at very intricate matters in (moral) epistemology. I do not claim to be able to answer these in a decisive manner. However, I do think that comparison to science, once more, is apt to spell trouble for moral realists because the antirealist defense in ethics is at least as promising as, if not more promising than, the one scientific antirealists have. Ultimately, to counter the second objection, antirealists need to make a plausible case that evidence transcendence is an unattractive position. Since it is arguably acceptable to be skeptical about knowledge in some domains but not others, antirealists don’t need to show that all kinds of evidence transcendence are implausible. They only need to do so *for the relevant domain*. In other words, moral antirealists need to cast doubt on the idea that the relevant facts about what makes moral theories correct could resist detection in principle.

In this regard, I propose that moral antirealists are in a better situation than antirealists in science. To see why, consider a simple example of evidence transcendence in the philosophy of science: the idea that, at a given time, there is either an even or an uneven number of stars in the universe, *no matter* whether we can ever know about it.^52^ This, I contend, would strike most people as at least initially plausible. Whether we know the correct number of stars in the universe seems unconnected to what that number is. Most laypeople and probably also most philosophers would be happy to acknowledge that the facts of the matter can and do transcend our knowledge in this case.

Yet our intuitions in other domains are often less optimistic. As a second example, could there be a fact of the matter about the meaning of words that is completely independent of what we know about those meanings?^53^ I’d wager that this sounds less plausible to most people. It is more difficult to imagine that there can be a fact of the matter to a word’s correct meaning, about which we as the speakers of that language could forever be in the dark.

What about evidence transcendence in ethics? The case in ethics strikes me as much closer to the latter scenario. To think that there is a truth about what makes acts right or wrong that could in principle evade our detection seems to be a dubious idea. This, I would argue following Wright, is ultimately due to two different forms of evidence transcendence being at play here.^54^ In the scientific case above, evidence transcendence is due to *contingencies of epistemic opportunity*: our measuring instruments or spatio-temporal situations not allowing us to attain knowledge about a case. In the described case in ethics, however, no such contingencies can be found, pointing to a more malign form of evidence transcendence. Although our moral explanations are clearly intimately related to our everyday lives, pertaining not to remote or otherwise inaccessible events, we would somehow be banned in principle from ever knowing which one is correct. This, although not impossible in principle, seems rather far-fetched.

Even if, as noted in the introduction, moral realists need not necessarily be committed to an epistemic position, that is, they need not claim that we can know moral truths, this comparison puts pressure on their view. They should at least be able to give a convincing account of why we would consistently fail to gain knowledge of the relevant sort. To claim that there are truths in some domain, but remain completely silent about why no such truths are known, is a rather uncomfortable stance. Especially if there are other domains where the lack of (secure) knowledge can be explained in a convincing way. Thus, if the above comparison is plausible, we can once more record that the antirealists’ case in ethics seems more promising than the antirealists’ case in science.

However, at this stage, one might suspect that the way I am framing matters unfairly stacks the deck against realists. As was noted in the Introduction, the dialectical situation in the philosophy of science is different from the one in ethics. In the philosophy of science, realists and antirealists agree that there are facts about, and we can thus have knowledge about, observables. They only disagree about whether this is also true for unobservables. Matters are different in metaethics. Moral realists only claim that there are *some* moral truths whereas antirealists in turn claim that there are none and we can therefore have no moral knowledge at all. Considering this, realists could simply accept that we cannot adjudicate between the rival theories, even grant that we may never be able to do so, but still think that the fact that these theories agree regarding their deontic upshots means that there is a fact of the matter at least about the deontic verdicts. They might even admit that there are no truths about what explains why some acts are right or wrong, but insist that some acts are nevertheless right or wrong. This finally brings us to the third objection, one that is distinctive of the moral variety of the skeptical argument.

### Objection III: A different dialectical situation?

Considering what has just been said, it seems like the underdetermination argument does not directly challenge moral realism at all, since it only challenges the explanatory but not the extensional claims of moral theories. One might be excused to think that it even strengthens moral realism. After all, according to (P1*), the best theories agree on extension. Shouldn’t this make us more confident that they do at least have the deontic side correct? Even if one grants that can never know the true ethical theory, the fact that all our best theories agree in their deontic results seems like strong evidence that at least these results are correct. Parfit and the consequentializers thus apparently leave us closer to the aim of finding some truths in ethics, which, on the standard view, is all the *moral* realist wants to claim.

I think that the antirealist reply should be two-pronged. First, antirealists should reject the claim that convergence between rival theories strengthens our entitlement in believing the verdicts those theories arrive at. For consider the following. Parfit and the consequentializers only show us that our theories converge in their deontic verdicts. Realists take this to reinforce the plausibility of those verdicts. Presumably, this is so because they now consider those verdicts to be doubly (or triply) supported.^55^ However there is a problem with this line of reasoning. For why are we supposed to believe that the fact that the rival theories agree upon those verdicts gives us reason to be more confident about the verdicts? After all, we know that they cannot all be true since they are still rivals. Yet support from false theories should not make us more confident in our verdicts. So the verdicts that the rival theories agree upon are not strengthened at all. The fact that a theory which we know to be false arrives at some result should not be considered an *additional* confirmation of that result, even if we think that this is the correct result.^56^

Note that antirealists need and should not claim that the underdetermination argument leads us to *lower* our credence in the particular verdicts. Indeed, that would threaten the whole argument, since the reason why we think that the rival theories are underdetermined is precisely because they account equally well for these verdicts. If underdetermination made us believe less in these verdicts, it would also impact the basis to believe in the phenomenon of underdetermination itself and the argument would thus be in danger of undermining itself. However, antirealists can still claim that the fact that the rival theories agree on what matters should not *increase* our credence, as people like Parfit thought. One upshot of the skeptical argument is thus to repudiate a seemingly promising way for moral realists to counter the argument from disagreement. It shows that convergence between the main traditions means nothing, as long as the explanatory disagreements are not resolved. This, although not a direct refutation of moral realism, is still a significant result and it indirectly betters the antirealist stance by taking one promising realist strategy of the table.

At this stage, realists could be tempted to withdraw to a disjunctive theory, e.g., a theory that claims that either acts are wrong because they are not maximizing utility, or because they are not consistent with the categorical imperative, or because they can consistently be rejected. But that seems unsatisfactory. It might be attractive if we were talking about only slightly different explanations, which we cannot adjudicate between for reasons such as our measuring instruments lacking in precision. We might, in such a case, still think that theories are close enough for our purposes and that we don’t need to make a decision. However, the case is different in ethics. As I have argued with reference to Dietrich and List and some of my earlier work, the main traditions of moral theorizing are not just slightly different. Instead, they offer mutually exclusive fundamental explanations of what makes acts right or wrong. It therefore seems more than disappointing to be told that we need not know which is true. As a comparison, consider the 16th century debate about planetary motion and imagine a disjunctive theory being offered that claimed to resolve the dispute by stating that either the earth or the sun is at the center of the solar system. It seems highly doubtful that, uttered as an alternative theory, this would have satisfied any scientist at the time and it also would not have alleviated the kinds of doubts that have prompted, e.g., Duhem’s skeptical arguments. I don’t think that ethicists should be satisfied with such an explanation either.

Yet I think that an even stronger conclusion should be drawn than this, which brings us to the second part of the antirealist answer. Let us for the sake of argument concede for a moment that the convergence on a set of verdicts makes it more plausible that those verdicts are also the correct ones. What has been won by that? Realists can now claim that there are at least some verdicts in ethics that we can have knowledge about. But is this enough? I don’t think so. Realists would in addition have to provide us with an explanation for two asymmetries.

First, they have to explain the asymmetry between ethics and science, where there is a corresponding discussion. Some philosophers of science have proposed that one can accept truths about the observable but not the unobservable. Most prominently, van Fraassen (1980) has argued that science doesn’t aim for more than empirically adequate theories. According to him, the goal of a scientific theory should not be to provide us with a true story of the unobservable features of the world. Instead, acceptance of a theory implies nothing more than believing its empirical predictions:

Science aims to give us theories which are empirically adequate; and acceptance of a theory involves as belief only that it is empirically adequate. (van Fraassen 1980, p. 12)

Yet, Van Fraassen is very clear that his *constructive empiricism* is in opposition to scientific realism and the position is typically considered to be an antirealist position. Its antirealism consist exactly in the fact that van Fraassen denies that we should believe what our scientific theories tell us beyond what we can already observe. The pressing realist questions, he argues, are not about observables but about unobservables. Scientific realists have mostly accepted this framing. They do not want to restrict their claims to what we can already observe, but urge us to also believe in the explanations our theories give us.

Realist in ethics might object that this does not threaten their position for the simple fact that realism might differ from domain to domain. Maybe realists in ethics can content themselves with knowing which acts are right or wrong and neglect the question of why this is so. This, I acknowledge, is not obviously implausible. It is not obvious that realism makes the same demands in all domains of human inquiry. Yet, I also think that the asymmetry would have to be explained. Otherwise, it seems ad hoc to simply claim that the structurally analogous position that gives rise to antirealism in science should be compatible with realism in ethics. Of course, realists might just thump the table and insist that according to the way the dispute has been defined so far, this is a realist position because it allows for some moral truths. They could claim that all that counts in ethics is deontic adequacy. Taking their inspiration from van Fraassen, they could call this new position *constructive deonticism*, insisting that, contrary to the structurally analogous position in science, constructive deonticism should count as a realist position. But that seems at least incurious. If we learn of a new position, and we learn that the analogous position in another domain has been considered to be squarely on the anti-realist side of the debate, we should wonder why it would count as a realist position in our domain. Realists should inquire into what attracted them to realism in the first place and whether what we are left with here, i.e., knowledge of what is right without any explanation of why it is right, is really all they want from a position that deserves the name of moral realism. My hunch is that most realists would not be satisfied with this.

We are, at this stage, reconsidering deep dialectical fault lines and inquiring into the definition and nature of moral realism itself. However, this seems appropriate since, traditionally, the distinction between (knowledge of) explanatory truths and (knowledge of) extensional truths has not played a role in the metaethical realism debate. It is only when we think of the matter in terms of underdetermination that this distinction really comes into view. This should prompt us to look at the debate in a new way. Maybe it was acceptable for realists to know some truths about clear cases (e.g. the wrongness of torturing people for the fun of it), while not knowing the truth about more convoluted cases (e.g. certain complicated trolley cases). However, to claim that we know what’s right and wrong, but have no idea what makes it so, is quite a different thing. Realists would have to explain to us this second asymmetry as well, that is, their asymmetrical reaction to extensional in contrast to explanatory disagreements. If disagreements about the deontic are indeed a threat to their position, as most realists acknowledge, what makes explanatory disagreements so special that they are not? To be sure, a fair definition of realism should not commit realists to hold that we know the answer to *every* moral question. However, can they indeed concede that a whole class of statements (those about moral explanation) are beyond our reach? Without further argument, that seems arbitrary. Usually, realists argue that when we are not able to find out the truth about a moral question, this can be explained, for example by citing such phenomena as vagueness. It would yet have to be shown why explanatory propositions in ethics summarily suffer from such an impairment. At least on first look, the explanations we put forward every day for why certain acts are right or wrong are to be taken at face value just like our claims about which acts are right or wrong. This presumably also holds for our moral theories. It thus seems arbitrary to claim that we need not know the truth about explanatory claims in order to be true realists.

Thus at the very least, the burden of proof is on realists to show why the new position (that we can know truths about the deontic but not the explanatory side of morality) is indeed a realist one. Barring further arguments, the idea of restricting the aim of our moral theories to deontic adequacy looks both ad-hoc and suspiciously similar to the one of restricting the aim of our scientific theories to empirical adequacy, which is, after all, an antirealist suggestion.

## Conclusion

Summing up, I have followed Dietrich and List in arguing that some recent developments in normative ethics are best interpreted using an idea from the philosophy of science: the underdetermination of theory by the evidence. The situation where the main traditions of moral theorizing agree on what we should do while at the same time disagreeing on why we should do so is structurally analogous to the the situation where some scientific theories agree on their extension while disagreeing when it comes to the explanations they put forward. Next, I have argued that this gives rise to a new skeptical challenge. I have proposed a standard form for that challenge, which is both simple and general. Finally, I have defended the argument against three objections, concluding that the prospects of antirealists in ethics vis-à-vis all three objections look at least as good as, if not better than, the prospects of antirealists in science. If the skeptical argument from underdetermination carries weight in science it should thus also do so in ethics. Convergence between our main moral traditions is not the good news some realists have been hoping for, but instead it lays the ground for a new skeptical challenge. This challenge might in the end even impact the way that the realism debate itself is framed, leading to a reassessment of what should count as a realist position in ethics.

## Footnotes

1 See Mackie (1977) for a classic statement of that argument.

2 Compare Dreier (1993) and, more recently, Portmore (2011). I will follow these authors in using *deontic* to refer only to the moral side of the deontic realm, not the domains of, e.g., the legal or of etiquette.

3 Together with the so-called *pessimistic metainduction*. Compare Stanford (2006, pp. 6–8) and Bortolotti (2008, pp. 98–104). Compare also Kukla (1998, p. 58) for the assessment that underdetermination is *undoubtedly the* biggest threat to scientific realism.

4 But compare Tersman (2019) for an attempt to argue from moral skepticism to moral antirealism.

5 Of course that does not mean that one accepts every contemporary scientific theory as true. But one does have to accept that the theoretical claims of scientific theories should be taken at face value.

6 Recent textbook examples include Tännsjö (2002) and Driver (2007).

7 For example Hare (1997), who suggests that Kant could have been a consequentialist.

8 As anyone familiar with the literature on so-called *trolley cases* will certainly agree.

9 In Volume 3 of OWM, Parfit argues that some versions of act-consequentialism might also be closer to these other traditions than often thought. However, since Parfit ultimately rejects act-consequentialism, for reasons too detailed to be repeated here, I will focus on his original arguments in Volume 1. See Parfit (2017, pp. 413–416 and pp. 433–435) for his reasons to reject act-consequentialism. Compare also Hooker (2020) for an excellent discussion of these reasons. Note that if Parfit’s rejection of act-consequentialism should turn out to be unfounded, that would further strengthen the challenge to the received view.

10 His goal, Parfit (2011a, pp. 338–339, pp. 369–370) informs us, is not to stay as true as possible to the original versions of those traditions, but instead to improve upon them.

11 See Parfit (2011a, pp. 377 ff.).

12 The term“consequentializing” goes back to Dreier (1993).

13 Parfit (2011a, p. 174).

14 Louise (2004) and Peterson (2013).

15 Dreier (2011).

16 Brown (2011).

17 Presumably precisely because one of the projects has been advertised as one of reconcilliaton, whereas the other is considered to be reductionist.

18 Physics provides for most of the standard examples of underdetermination. Examples include the rivalry between Copernicus’ new theory of planetary motion and its Ptolemaic alternative as well as the one between corpuscular and wave theories of light.

19 For example by Portmore (2011, p. 109) and Suikkanen (2014, p. 104).

20 Compare Baumann (2018, pp. 197–198) and Baumann (2019, pp. 520–521).

21 Compare Baumann (2018, pp. 200–201) for a discussion of their incompatibility.

22 With the exception of mentioning how a parallel view to scientific instrumentalism in ethics might clash with their reason-based representation. Compare Dietrich and List (2017, p. 425). However, they don’t pursue this line of thinking any further.

23 Parfit (2011b, p. 543 ff.).

24 As I (Baumann 2018, p. 217) note, the argument is not to be understood as a general argument against moral realism. This is also true for Baumann (2021a), which uses the underdetermination argument as part of a dilemma for Parfit.

25 Compare Bortolotti (2008) and Stanford (2017).

26 Compare Kukla (1998, p. 58), Psillos (1999, p. 164), and Douven (2008, pp. 294–295).

27 This does not mean that one of the theories has to include an explicit negation of one of the others’ claims. However, *pace* Quine (1975, p. 327), it does require more than us being contingently unable to reconcile their predicates.

28 See the title of Kukla (2001). The longer explanation for why the premise is uninterestingly true involves the idea that it only concerns *deductive* instead of *ampliative* underdetermination and that the former is easy to establish because deductive logics is not restrictive enough to exclude certain obviously implausible theories. Compare Laudan (1990) for this point.

29 For example Bortolotti (2008).

30 The opposite could be true as well: the skeptical argument from underdetermination might fail in science, but nevertheless prove successful in ethics. However, since I’m not evaluating its validity in science, I won’t look into this third possibility.

31 Compare Baumann (2018, p. 207, 2019, pp. 518–519).

32 Compare Boyd (1988, pp. 184–185) and Audi (2008, p. 476).

33 Compare Smith (2010, pp. 83–84) for the assessment that verdicts and judgements are less controversial notions in this context.

34 Douven (2008, p. 293).

35 It might be applicable on some naturalist conceptions, but I do not want to presuppose these.

36 See Ladyman (2002) and Stanford (2017).

37 Most prominently, arguably, Derek Parfit. Compare Parfit (2011b, pp. 486 ff., 2017, p. 59) and the discussion in Baumann (2021a). I gloss over the fact that Parfit eschews the label *realism*, preferring *cognitivism* instead, since Parfit clearly accepts that there are true claims in ethics of which we can have knowledge, which is the position that is being attacked by the skeptical argument.

38 Park (2009, pp. 116–119) provides a good overview of the different positions the skeptical argument from underdetermination has been thought to entail.

39 An especially forceful outline of this objection can be found in Laudan (1990).

40 van Fraassen (1980, pp 87–96).

41 Tulodziecki (2012, p. 326).

42 Hooker (2000) offers a very influential line of reasoning for Rule Consequentialism along these lines.

43 In Baumann (2018, pp. 210–211) I consider both strategies but remain neutral because I do not think that Parfit could make use of Objection I in any case.

44 There is also an additional worry about this strategy. For if nothing beyond extension is relevant to the truth of a theory, then how is this compatible with the claim in the first premise that we are facing rival theories? If the theories have the same extension, and this is all that matters for truth, then the theories seem not to be in conflict anymore. Of course there would still be different ways to organize the evidence. But the theories would no longer be *rival* in the outlined sense. I will take up discussion of this issue in Sect. 4.3, since it ties in with one initially promising realist reply that needs a more thorough treatment than I can offer at this stage.

45 This is sometimes called the *completeness axiom*. Compare Brandt (1959, p. 295). It is not universally accepted, but it has seemed plausible to many. Note that this does not mean that moral theories give verdicts of rightness or wrongness for every particular case. There might be cases where there is no right or wrong. But it does mean that, so far as there are correct deontic verdicts for specific cases, moral theories do in principle cover them all. Remember also that I understand *deontic* to refer only to the moral domain, not the domains of the legal or of etiquette.

46 Schroeder (2017, p. 1479).

47 See Emet (2010). As an anonymous referee has pointed out to me, it is interesting that whereas philosophers of science focus more on formal virtues like simplicity, etc., it is more common in ethics to directly discount theories because one finds their reasons structure independently implausible. This does not impact the general point here, however.

48 Compare Baumann (2021b, pp. 474–476) for a more detailed discussion of this point.

49 On this point see also Baumann (2019, p. 524).

50 Compare for example Nebel (2012) for a discussion of the modal strength of Parfit’s convergence claim.

51 Quine (1975, pp. 327 ff.) considers this reaction, but wavers between it and a more straightforward answer, according to which we are entitled to hold on to the theory we originally held. A very informative discussion of what has been called the *ignorance* and the *arrogance* responses to underdetermination is provided by Newton-Smith (1978) and Bergström (1984). See also Sklar (1975, pp. 378 ff.) for an argument that it is rational to react to cases of underdetermination on the basis of a principle of methodological conservatism, which prescribes to stick with the original theory one accepts or the one more continuous with pre-existing theories.

52 For this line of reasoning see Baumann (2021b, pp. 476–477).

53 The example of course alludes to Quine’s famous thesis about the *indeterminacy of translation*. See, for example, Quine (1960, pp. 26 ff.).

54 Wright (1992, pp. 151–152, 2000, pp. 360–361).

55 As in Parfit’s famous *Triple Theory*.

56 As I (Baumann 2021a, p. 1016) argue with regard to Parfit’s converging theories, it is highly misleading to talk of *triply supported* theories, if one does not accept the explanatory claims of two of the three theories.
